# Research Literacy and Research Engagement Following Project‐Embedded Support Among Clinical Nurses: An Exploratory Nonrandomised Controlled Study

**DOI:** 10.1155/jonm/6444368

**Published:** 2026-10-21

**Authors:** Qian Li, Ailing Ni

**Affiliations:** ^1^ Nursing Department Affiliated People’s Hospital of Jiangsu University Zhenjiang Jiangsu China ujs.edu.cn; ^2^ Changzhou No. 2 People’s Hospital Changzhou Jiangsu China czey.com

**Keywords:** clinical nursing, mentoring, research capacity, research engagement, research literacy

## Abstract

**Aim:**

To explore whether sustained, multicomponent, project‐embedded support was associated with changes in clinical nurses’ research literacy, research translation capability and research engagement.

**Background:**

One‐off research training may improve knowledge but often provides limited support for moving clinical questions into sustained research action.

**Methods:**

An exploratory nonrandomised controlled pretest–posttest study was conducted in a tertiary teaching hospital in eastern China. Forty‐four nurses were analysed (intervention *n* = 19; concurrent comparison *n* = 25). The intervention combined structured research activities, an ongoing research clinic, project tracking and individualised support. The four core outcomes were functional research literacy, interactive research literacy, critical research literacy and research translation capability. Theory‐informed secondary outcomes related to research opportunity, motivation and behaviour were also assessed and objective project progression was described. Baseline‐adjusted analysis of covariance (ANCOVA) was used as the main adjusted analysis, with a sensitivity model additionally adjusting for postgraduate education and senior professional title.

**Results:**

Interactive research literacy was the only core outcome with a significant unadjusted time × group interaction. In baseline‐adjusted ANCOVA, all four core outcomes favoured the intervention after false discovery rate correction; after additional adjustment, only interactive research literacy remained statistically significant (adjusted difference 0.348, 95% CI 0.113–0.584; *p* = 0.005). Objective project milestones were observed in the intervention group but were not available for between‐group comparison.

**Conclusions:**

Project‐embedded support was associated most consistently with interactive research literacy. Findings for the other core domains were less robust after additional covariate adjustment. Given the nonrandom allocation, small sample and preliminary outcome measure, the findings should be interpreted as exploratory.

**Implications for Nursing Management:**

Nurse managers may consider longitudinal structures that connect research learning with real clinical projects, repeated dialogue, methodological support and project follow‐up, while evaluating such programmes with longer follow‐up and stronger comparative designs.

## 1. Introduction

Clinical nurses are well positioned to identify problems that affect patient care, workflow and quality, but identifying a clinical problem does not necessarily mean that it can be translated into a researchable question or sustained project. Reviews of clinical nurses’ research capacity describe uneven research knowledge and skills together with recurring barriers involving time, mentorship, organisational support and access to research resources [1–3]. These constraints can affect study design, ethics, data collection, analysis, writing and progression from an initial idea to active research.

Previous efforts to strengthen nurses’ research capacity have included lectures, short courses, workshops, mentoring and project‐based programmes. These approaches can improve proximal outcomes such as knowledge and confidence, but transfer into active research work is less certain. A systematic review found that interactive and activity‐based approaches were more promising than passive teaching for improving nurses’ research literacy [4]. A later scoping review found that many research‐education programmes assessed knowledge, confidence or satisfaction, while relatively few tracked protocol completion, manuscripts or organisational outcomes [5]. That review focused on programmes delivered in high‐income countries, also leaving uncertainty about transferability across healthcare systems and organisational contexts. More recent reviews have therefore argued for context‐sensitive programmes that combine individual capability building with leadership, mentorship and research infrastructure [3, 6].

Two related gaps therefore remain: whether gains in research knowledge and capability translate into sustained research engagement and progression through real clinical projects, and whether longitudinal support can be organised in ways that remain meaningful across different organisational contexts. A preceding qualitative study in Chinese clinical nurses showed that research engagement could stall at evidence interpretation, research dialogue, problem translation or project implementation, particularly when hierarchy, psychological safety, workload or continuity of support were unfavourable [7]. These findings informed the development of the present intervention.

This study used two complementary perspectives. First, Nutbeam’s functional, interactive and critical literacy framework was conceptually adapted to clinical research literacy [8]. Functional research literacy concerns understanding research information; interactive research literacy concerns discussing evidence and research ideas with others; and critical research literacy concerns judging credibility, clinical relevance and feasibility. Second, the capability–opportunity–motivation–behaviour (COM‐B) model was used to consider the conditions under which these capacities may translate into research behaviour [9]. We therefore developed a sustained, multicomponent, project‐embedded support programme that linked research learning to participants’ own clinical projects. This exploratory study examined whether the programme was associated with changes in functional, interactive and critical research literacy and research translation capability, while also describing theory‐informed indicators of research engagement and objective project progression. Because allocation was nonrandom, the analyses were intended to estimate adjusted associations rather than establish causal effects.

## 2. Methods

### 2.1. Design and Setting

An exploratory nonrandomised controlled pretest–posttest quasiexperimental study with a concurrent comparison group was conducted from January to August 2026 in a tertiary teaching hospital in eastern China. Baseline assessment was completed on 3 January, before project review and structured support. Project review and group allocation occurred on 8 January. Postintervention assessment was completed on 18 August, after the planned intervention activities.

### 2.2. Participants and Allocation

Participants were eligible if they were clinical nurses who voluntarily joined the hospital nursing research programme, submitted a clinical research topic or project proposal before formal project review and completed the baseline assessment. The recruitment notice was open to clinical nurses across the hospital. No additional exclusion criteria based on age, clinical specialty, professional title or educational level were applied. Submitted projects were formally scored and ranked by four doctoral‐level reviewers: two nursing experts, one clinical physician and one hospital research‐administration supervisor. Criteria included scientific merit, innovation and feasibility. Nurses whose projects were selected for the annual structured support programme formed the intervention group. Nurses who completed baseline assessment and submitted a proposal but were not selected formed the concurrent comparison group.

Allocation was determined by project review rather than randomisation. Both groups had already expressed research interest at baseline. Although recruitment was open to clinical nurses across the hospital, enrolment required voluntary participation and submission of a research topic or project proposal. The cohort was therefore self‐selected for research interest and should not be regarded as representative of research‐naïve clinical nurses. Prior research‐methodology training was not quantified as a standardised baseline variable because research exposure could have been acquired through heterogeneous formal and informal routes. Because scientific merit, innovation and feasibility were part of the selection process, allocation could also reflect differences in project maturity or research readiness that were not fully captured by measured participant characteristics. Project‐review scores were not included in the analytic models; residual confounding by project quality therefore remains possible. The study used the complete eligible annual cohort; the sample size therefore reflected programme size rather than an a priori power calculation. The final analytic sample comprised 44 nurses with matched baseline and postintervention data: 19 in the intervention group and 25 in the concurrent comparison group. Given the small, nonrandomised cohort, effect estimates and confidence intervals (CIs) were interpreted as exploratory.

### 2.3. Conceptual Framework and Intervention Development

Research literacy and COM‐B were used as complementary, not one‐to‐one, frameworks. Research literacy defined capacities that the programme sought to develop. COM‐B identified conditions that could support translation of these capacities into research behaviour. The intervention was also informed by a preceding qualitative study conducted independently before the structured programme began [7]. The conceptual framework is shown in Figure 1, and the theory‐to‐intervention mapping is provided in Supporting Information Table S1.

**FIGURE 1:**
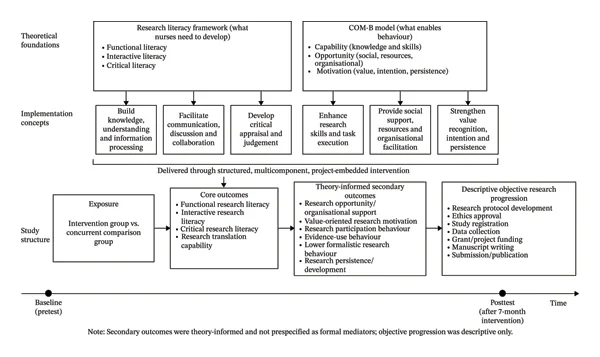
Conceptual framework linking theoretical foundations, intervention design principles and study outcomes.

The programme was designed around five practical targets: understanding research, participating in research dialogue, making critical judgements, translating clinical problems into research tasks and sustaining project progression. A single intervention component could address more than one theoretical construct.

### 2.4. Intervention Delivery

The intervention ran from January to 18 August 2026 and combined three components: structured core activities, an ongoing research clinic and project‐stage tracking with individualised support (Figure 2).

**FIGURE 2:**
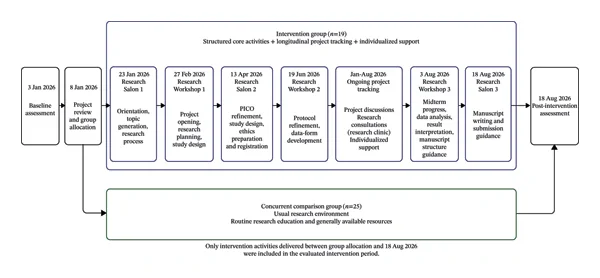
Intervention delivery and study timeline.

Structured core activities comprised three research salons, defined as interactive research seminars, and three research workshops/project meetings. Early activities focused on clinical problem identification, topic refinement and study design. Middle activities addressed population, intervention, comparison and outcome (PICO) refinement, feasibility, ethics, registration and protocol development. Later activities addressed data management, analysis, interpretation, manuscript structure, writing and submission. The salons were held on 23 January, 13 April and 18 August and lasted approximately 1.5 h each. Workshops were held on 27 February, 19 June and 3 August and lasted approximately 4 h each. All 19 intervention participants attended all six core activities.

The structured activities were delivered by a PhD‐prepared nurse researcher who oversaw nursing research within the Nursing Department. The research clinic remained available throughout the programme and provided project‐specific support for question refinement, design, ethics, registration, data management, statistical analysis, writing and grant applications. Forty‐seven research‐clinic consultations were recorded. Project progress was reviewed longitudinally, and the content and frequency of individual support were tailored to project stage and barriers.

### 2.5. Concurrent Comparison Condition

Comparison participants worked in the same hospital research environment. They retained access to routine research education, hospital‐wide open activities and general research resources. They could also seek procedural advice from the hospital research administration on ethics, registration and other research processes and some participants pursued these steps through self‐directed learning. They did not receive the structured longitudinal project‐tracking programme or intervention‐specific individualised research‐clinic support. The comparison condition was therefore an active usual research environment rather than a no‐intervention condition.

### 2.6. Outcomes and Measurement

Research‐related outcomes were assessed at baseline and postintervention using the Clinical Research and Practice Literacy–Behavior Scale for Nurses (CRPL‐BSN), an investigator‐developed, theory‐informed questionnaire for the clinical nursing research context. The instrument was not adapted from a single established scale. Item content was generated from the research‐literacy framework, COM‐B and the preceding qualitative findings.

The initial item pool underwent content review by six nursing experts, each with at least a master’s degree and an associate senior professional title or above. The reported item‐level content validity index (I‐CVI) was 0.83 and the scale‐level content validity index, average method (S‐CVI/Ave), was 0.92. Three clinical nurses then pilot‐tested the questionnaire and provided feedback on wording and comprehensibility. The instrument has not undergone independent large‐sample structural validation, including exploratory or confirmatory factor analysis, and construct validity remains to be established. It was therefore treated as a study‐specific, theory‐informed measure rather than a fully validated scale. Internal consistency was evaluated in the present sample. Detailed domain scoring is provided in Supporting Information Table S2.

Four core outcomes were specified because they were the programme’s most direct capability targets: functional research literacy, interactive research literacy, critical research literacy and research translation capability. Because the factor structure and construct validity of the CRPL‐BSN have not yet been established in an independent sample, these domain scores were interpreted cautiously as preliminary, theory‐informed measures.

Theory‐informed secondary outcomes were research opportunity/organisational support, value‐oriented research motivation, research participation behaviour, evidence‐use behaviour, lower formalistic research behaviour and research persistence/development. These were treated as secondary outcomes rather than formal mediators because only two measurement occasions were available.

### 2.7. Objective Research Progression

Objective progression was recorded for intervention participants using longitudinal project‐tracking records and, where applicable, institutional research records. Participant‐level milestones were protocol completion, ethics application and approval, study registration, commencement of data collection, manuscript writing and manuscript submission. Government funding was counted at the project level, and publication/acceptance at the manuscript level. Funding and manuscript milestones were restricted to outcomes occurring within the nursing research group between February and August 2026. Because comparable objective progression data were unavailable for the comparison group, these indicators were descriptive programme‐associated milestones and were not used to estimate intervention effectiveness.

### 2.8. Data Collection and Statistical Analysis

Baseline and postintervention records were matched using participant identifiers and then de‐identified. All 44 participants had complete matched data. The individual nurse was the unit of assignment and the unit of analysis. Cronbach’s alpha was calculated separately at baseline and postintervention. Change‐score distributions were assessed with Shapiro–Wilk tests.

Baseline continuous variables were compared using independent‐samples *t* tests or Welch’s *t* test when variance homogeneity was not met. Categorical variables were compared with Fisher’s exact test. Standardised mean differences (SMDs) were also reported because allocation was nonrandomised.

Within‐group changes were examined using paired‐samples *t* tests and summarised with mean change, 95% CIs and Cohen’s dz. Unadjusted between‐group effects were examined with a 2 × 2 mixed‐design repeated‐measures analysis; the time × group interaction represented the difference in change between groups.

The main adjusted analysis used analysis of covariance (ANCOVA) with the postintervention score as the dependent variable, group as the fixed factor and the corresponding baseline score as a covariate. Homogeneity of regression slopes was assessed using the group × baseline interaction. Adjusted mean differences, 95% CIs, *p* values and partial *η* ^2^ were reported. A sensitivity model additionally adjusted for postgraduate education and senior professional title, which showed the clearest baseline imbalance. Benjamini–Hochberg false discovery rate (FDR) correction was applied to the four core outcomes from the main ANCOVA. Given the exploratory design and small cohort, emphasis was placed on the direction and precision of effect estimates rather than statistical significance alone. Analyses used IBM SPSS Statistics 26.0, with two‐sided *p* < 0.05.

### 2.9. Ethics and Reporting

The study was approved by the Ethics Committee of the Affiliated People’s Hospital of Jiangsu University (Approval No. [2025] KYW001‐92) and conducted in accordance with the Declaration of Helsinki. All participants provided written informed consent. Participants and programme staff were not blinded to allocation because participation in the structured support programme was evident. Intervention delivery was documented through attendance records, research‐clinic logs and longitudinal project‐tracking records. No clinical adverse‐event outcome was prespecified because the intervention consisted of educational and research‐support activities. Reporting followed the Transparent Reporting of Evaluations with Nonrandomized Designs (TREND) statement for nonrandomised controlled evaluations, and intervention description was organised using Template for Intervention Description and Replication (TIDieR) principles [10, 11].

## 3. Results

### 3.1. Participant Characteristics and Measurement Performance

All 44 participants completed both assessments (Figure 3). No conventional baseline comparison reached statistical significance, but SMDs indicated meaningful imbalance in postgraduate education, senior professional title and several research‐related outcomes (Table 1). The intervention group generally had higher baseline research‐related scores.

**FIGURE 3:**
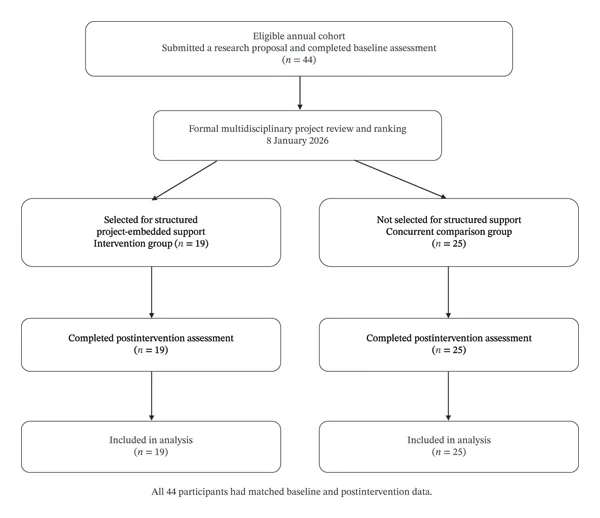
Participant flow through allocation, follow‐up and analysis.

**TABLE 1:** Baseline characteristics and baseline outcome scores.

Variable	Intervention (*n* = 19)	Concurrent comparison (*n* = 25)	Test	Statistic	*p* value	SMD
*Participant characteristics*						
Age, years	38.37 ± 5.93	36.72 ± 4.89	Independent *t*	1.011	0.318	0.30
Clinical experience, years	16.32 ± 7.48	13.52 ± 6.43	Independent *t*	1.332	0.190	0.40
Postgraduate degree, *n* (%)	3 (15.8)	10 (40.0)	Fisher’s exact	—	0.105	−0.56
Associate chief/chief nurse, *n* (%)	12 (63.2)	9 (36.0)	Fisher’s exact	—	0.127	0.56
Initial education ≥ bachelor, *n* (%)	9 (47.4)	15 (60.0)	Fisher’s exact	—	0.543	−0.26

*Baseline outcome scores*						
Functional research literacy	3.821 ± 0.552	3.660 ± 0.561	Independent *t*	0.950	0.348	0.29
Interactive research literacy	3.888 ± 0.582	3.695 ± 0.586	Independent *t*	1.086	0.284	0.33
Critical research literacy	4.072 ± 0.514	3.810 ± 0.484	Independent *t*	1.733	0.090	0.53
Research translation capability	3.699 ± 0.508	3.371 ± 0.565	Independent *t*	1.989	0.053	0.61
Research opportunity/support	3.932 ± 0.590	3.617 ± 0.520	Independent *t*	1.880	0.067	0.57
Value‐oriented research motivation	4.400 ± 0.490	4.128 ± 0.586	Independent *t*	1.635	0.110	0.50
Research participation behaviour	4.167 ± 0.692	3.727 ± 0.992	Independent *t*	1.651	0.106	0.51
Evidence‐use behaviour	4.200 ± 0.622	3.936 ± 0.759	Independent *t*	1.233	0.224	0.38
Lower formalistic research behaviour	3.284 ± 1.108	3.048 ± 0.669	Welch *t*	0.822	0.418	0.26
Research persistence/development	4.432 ± 0.607	4.200 ± 0.580	Independent *t*	1.286	0.205	0.39

*Note:* Continuous variables are presented as mean ± SD and categorical variables as *n* (%). Independent‐samples *t* tests were used for continuous variables; Welch’s *t* test was used when the homogeneity‐of‐variance assumption was not met. Categorical variables were compared using Fisher’s exact test. The sign indicates the direction of the difference (intervention minus concurrent comparison), and the absolute value reflects the magnitude of baseline imbalance. Higher outcome scores indicate more favourable levels.

Abbreviation: SMD = standardised mean difference.

Internal consistency was acceptable to high for most domains. Cronbach’s alpha ranged from 0.736 to 0.924 at baseline and from 0.691 to 0.938 postintervention; critical research literacy at postintervention was marginally below 0.70. Change‐score distributions showed no significant departure from normality (Supporting Information Table S3).

### 3.2. Pre–Post and Unadjusted Effects

Both groups improved in several research literacy and capability outcomes (Table 2). Interactive research literacy was the only core outcome with a significant unadjusted time × group interaction. Its net difference in change was 0.327 points (*p* = 0.048; partial *η* ^2^ = 0.090). No significant time × group interaction was observed for the other three core outcomes or for the theory‐informed secondary outcomes.

**TABLE 2:** Pre–post changes and unadjusted group‐by‐time effects.

Outcome	Intervention group (*n* = 19)					Concurrent comparison group (*n* = 25)					Between‐group effect		
	Pre	Post	Δ (95% CI)	Within‐group *p*	Cohen’s dz	Pre	Post	Δ (95% CI)	Within‐group *p*	Cohen’s dz	Net Δ	Time × group *p*	Partial *η* ^2^
Functional research literacy	3.821 ± 0.552	4.226 ± 0.346	0.405 (0.083, 0.728)	0.017	0.61	3.660 ± 0.561	3.920 ± 0.381	0.260 (0.056, 0.464)	0.015	0.53	0.145	0.412	0.016
Interactive research literacy	3.888 ± 0.582	4.395 ± 0.443	0.507 (0.212, 0.801)	0.002	0.83	3.695 ± 0.586	3.875 ± 0.427	0.180 (−0.008, 0.368)	0.060	0.39	0.327	0.048	0.090
Critical research literacy	4.072 ± 0.514	4.395 ± 0.405	0.322 (0.017, 0.628)	0.040	0.51	3.810 ± 0.484	4.000 ± 0.389	0.190 (0.000, 0.380)	0.050	0.41	0.132	0.426	0.015
Research translation capability	3.699 ± 0.508	4.143 ± 0.524	0.444 (0.117, 0.770)	0.010	0.66	3.371 ± 0.565	3.686 ± 0.378	0.314 (0.136, 0.493)	0.001	0.73	0.129	0.445	0.014
Research opportunity/support	3.932 ± 0.590	4.120 ± 0.479	0.188 (−0.093, 0.469)	0.177	0.32	3.617 ± 0.520	3.800 ± 0.602	0.183 (−0.092, 0.458)	0.182	0.27	0.005	0.979	< 0.001
Value‐oriented research motivation	4.400 ± 0.490	4.611 ± 0.434	0.211 (−0.105, 0.526)	0.178	0.32	4.128 ± 0.586	4.296 ± 0.477	0.168 (−0.139, 0.475)	0.270	0.23	0.043	0.844	0.001
Research participation behaviour	4.167 ± 0.692	4.675 ± 0.551	0.509 (0.152, 0.865)	0.008	0.69	3.727 ± 0.992	4.053 ± 0.836	0.327 (−0.046, 0.699)	0.083	0.36	0.182	0.478	0.012
Evidence‐use behaviour	4.200 ± 0.622	4.621 ± 0.432	0.421 (0.105, 0.737)	0.012	0.64	3.936 ± 0.759	4.160 ± 0.656	0.224 (0.000, 0.448)	0.050	0.41	0.197	0.282	0.028
Lower formalistic research behaviour	3.284 ± 1.108	3.789 ± 0.790	0.505 (−0.061, 1.071)	0.077	0.43	3.048 ± 0.669	3.224 ± 1.043	0.176 (−0.289, 0.641)	0.443	0.16	0.329	0.351	0.021
Research persistence/development	4.432 ± 0.607	4.737 ± 0.313	0.305 (−0.047, 0.657)	0.085	0.42	4.200 ± 0.580	4.296 ± 0.578	0.096 (−0.191, 0.383)	0.496	0.14	0.209	0.338	0.022

*Note:* Δ = postintervention minus baseline. Within‐group changes were assessed using paired‐samples *t* tests. Cohen’s dz is the standardised within‐group change. Net Δ = intervention‐group change minus concurrent‐comparison‐group change. The time × group *p* value represents the unadjusted between‐group difference in change. Positive net Δ values favour the intervention group.

### 3.3. Adjusted Core Outcomes

In the main baseline‐adjusted ANCOVA, all four core outcomes favoured the intervention group and remained significant after FDR correction (Table 3). The largest adjusted difference was observed for interactive research literacy (0.449, 95% CI 0.211–0.687; partial *η* ^2^ = 0.261; *q* = 0.002). Functional research literacy, critical research literacy and research translation capability also met the FDR‐adjusted threshold (all *q* = 0.015). These estimates should be interpreted in the context of nonrandom allocation and baseline imbalance.

**TABLE 3:** Adjusted between‐group differences in core outcomes.

Outcome	Main ANCOVA adjusted difference (95% CI)	*p* value	Partial *η* ^2^	FDR q	Sensitivity model adjusted difference (95% CI)	*p* value	Partial *η* ^2^
Functional research literacy	0.277 (0.056, 0.498)	0.015	0.135	0.015	0.226 (−0.004, 0.455)	0.054	0.092
Interactive research literacy	0.449 (0.211, 0.687)	< 0.001	0.261	0.002	0.348 (0.113, 0.584)	0.005	0.187
Critical research literacy	0.336 (0.091, 0.580)	0.008	0.158	0.015	0.209 (−0.052, 0.470)	0.113	0.063
Research translation capability	0.351 (0.084, 0.618)	0.011	0.147	0.015	0.222 (−0.051, 0.494)	0.108	0.065

*Note:* The main ANCOVA adjusted for the corresponding baseline outcome score. The sensitivity model additionally adjusted for postgraduate education and senior professional title. Adjusted differences represent intervention minus concurrent comparison. Benjamini–Hochberg FDR correction was applied to the four core outcomes. The group × baseline interaction was nonsignificant for all four core outcomes, supporting the homogeneity‐of‐regression‐slopes assumption.

In the sensitivity model, additionally adjusting for postgraduate education and senior professional title, effect estimates were attenuated. Interactive research literacy remained statistically significant (adjusted difference 0.348, 95% CI 0.113–0.584; *p* = 0.005), whereas functional research literacy, critical research literacy and research translation capability no longer reached conventional statistical significance. Interactive research literacy therefore showed the greatest robustness to additional covariate adjustment.

### 3.4. Theory‐Informed Secondary Outcomes

In the main baseline‐adjusted analyses, value‐oriented research motivation, research participation, evidence use and research persistence/development favoured the intervention group (Supporting Information Table S4). Research opportunity/support and lower formalistic research behaviour did not differ significantly. None of the secondary outcomes remained statistically significant after additional adjustment for postgraduate education and senior professional title; these findings were therefore treated as exploratory and hypothesis‐generating.

### 3.5. Descriptive Objective Research Progression

Within the intervention group, all 19 participants completed research protocols and submitted ethics applications that were approved. Eighteen (94.7%) completed study registration and commenced data collection. Eleven (57.9%) began manuscript writing and nine (47.4%) submitted a manuscript. Ten participant‐associated projects received government funding or grants, and two manuscripts in Science Citation Index (SCI)‐indexed journals were published or accepted during February–August 2026 (Supporting Information Table S5). These data describe progression within the supported cohort; because comparable milestones were not collected for the comparison group, no between‐group effect can be inferred.

## 4. Discussion

### 4.1. Principal Findings

The clearest finding was the pattern for interactive research literacy. It was the only core outcome with a significant unadjusted time × group interaction and it remained statistically significant after additional adjustment for postgraduate education and senior professional title. Baseline‐adjusted analyses also suggested favourable differences in functional and critical research literacy and research translation capability, but these estimates were attenuated in the sensitivity model. Given the allocation mechanism, small sample and preliminary measurement tool, the results are better read as exploratory evidence of association than as confirmation of programme efficacy.

The intervention was intended to change more than research knowledge. Reviews show that research‐capacity programmes often improve knowledge or confidence, yet many studies stop short of asking whether nurses can use those gains in active research work [4, 5]. More recent work places greater emphasis on the interaction between individual capability and organisational support, rather than on education alone [3, 6].

### 4.2. Interactive Research Literacy as a Link Between Knowledge and Action

Interactive research literacy was repeatedly practised throughout the programme. Participants presented clinical problems, explained research decisions, received peer and expert feedback and returned with revised project work. The research clinic created further opportunities for one‐to‐one discussion. Research learning therefore occurred through dialogue around real tasks.

This interpretation is consistent with our earlier qualitative findings, in which nurses described research engagement as being influenced by professional hierarchy, psychological safety and their ability to bring research ideas into team discussion [7]. O’Brien et al. [12] similarly showed that accessible nurse leadership and reciprocal trust could support clinical nurses’ involvement in research. Project‐based programmes have also highlighted peer collaboration, academic support, experiential learning and clinical ownership as important conditions for developing research capacity [13, 14]. Recent reviews reinforce that research engagement depends not only on individual knowledge and skills but also on mentorship, leadership, research infrastructure and organisational support [3, 6].

Within the present programme, these conditions provide a plausible explanation for why interactive research literacy showed the most consistent between‐group signal. Peer discussion repeatedly required nurses to articulate research questions, justify methodological decisions and respond to feedback, thereby providing both practice and social opportunity for research dialogue. Project‐specific academic and methodological support may have strengthened capability by making guidance available when concrete research decisions were required, rather than separating research education from its application. Working on questions derived from nurses’ own clinical practice may also have reinforced motivation by maintaining the relevance and ownership of the research task. Viewed through COM‐B, these features may have worked together to support movement from understanding research to participating more actively in the research process. However, these pathways are theoretically plausible explanations rather than confirmed mediating mechanisms, because the present study was not designed to test mediation.

### 4.3. Effects on Other Core Outcomes and Improvement in Both Groups

Functional research literacy, critical research literacy and research translation capability also favoured the intervention in the main baseline‐adjusted analysis. Timing probably mattered. Participants received design support while developing protocols, ethics and registration guidance when projects were starting, and analysis and writing support later. The learning was tied to a task that needed to be completed at that point, rather than delivered long before it could be used. This task‐linked approach is consistent with research‐capacity programmes that embed learning in ongoing projects and combine it with mentorship and accessible research support [5, 6].

The comparison group also improved, and that finding is important. These nurses were not research‐naive or uninterested in research. They had voluntarily entered the hospital programme and submitted a research proposal at baseline, so they already had a reason to learn. During follow‐up, they could study independently, use routine hospital resources and seek procedural advice from the hospital research administration about ethics, registration and other research processes. The comparison condition was therefore active, not an absence of research support.

Improvement in both groups is understandable in that context. Motivation, self‐directed learning and access to routine expertise can all move a project forward, even without a structured programme. Reviews of nursing research capacity similarly describe research engagement as the product of individual motivation, access to expertise and the surrounding organisational environment [2, 3]. The relevant question in this study was therefore whether structured, longitudinal and project‐embedded support added something beyond an already research‐active institutional environment.

The active comparison may have reduced the contrast attributable to structured support because both groups could continue to learn and seek routine institutional advice. However, selection into the intervention group could have operated in the opposite direction by favouring more mature projects or more research‐ready nurses. The net direction of bias therefore cannot be determined. Interactive research literacy nevertheless remained the clearest between‐group signal. Repeated project discussion, feedback and follow‐up may have provided an advantage that self‐directed learning or occasional procedural consultation could not offer as consistently.

The attenuation of the other three core outcomes after additional adjustment is important. It indicates that the apparent advantages in these domains were sensitive to covariate choice and should not be interpreted as robust intervention effects. The small sample also produced relatively imprecise estimates. By contrast, interactive research literacy was less sensitive to these uncertainties and remained the most consistent signal across analyses.

### 4.4. Secondary Outcomes and Organisational Conditions

Secondary outcomes were less consistent than the core outcomes. In the main model, value‐oriented motivation, research participation, evidence use and research persistence favoured the intervention group. After additional adjustment for education and professional title, however, these associations weakened. Part of that pattern may reflect differences in prior academic preparation and professional position, both of which can shape how easily nurses engage with research opportunities. These secondary findings should therefore be viewed as hypothesis‐generating rather than as established programme effects.

COM‐B helps separate what the programme could change from what remained outside its reach. Capability was addressed directly through teaching, feedback, consultation and repeated project work. Opportunity was more complicated. The programme increased social and methodological opportunities by giving nurses access to mentors, peers and research consultation. It did not change staffing levels, routine workload or protected research time. Reviews repeatedly identify these organisational conditions, together with leadership and research culture, as important determinants of clinical nurses’ research engagement [2, 3].

This distinction may explain why perceived opportunity/support showed little additional change. Participants had better access to advice, but most still had to conduct research alongside their usual clinical duties. The programme changed how support was organised; it did not remove the practical constraints of doing research in a busy service. O’Brien et al. [12] reported a similar tension: supportive leadership and trust encouraged research participation, while organisational barriers still shaped what nurses were able to do.

Motivation also needs a cautious reading. Both groups entered the study voluntarily and had already submitted proposals, so motivation was high from the start. There was limited room for scores to rise. Real project work can also make the demands of research more visible. Ethics review, recruitment, data collection and publication take time and persistence. A stable motivation score can therefore sit alongside increasing research activity; it does not necessarily mean that engagement failed to develop.

Research participation, evidence use and persistence are also more distal than research literacy. They need time, repeated opportunities and an environment in which new skills can actually be used. Seven months was long enough to observe changes in research capability and substantial project progression, but it may be too short to expect stable change in broader research behaviour. For nurse managers, the implication is practical: capability building is a starting point, not the whole solution. Long‐term gains are more likely when project‐embedded support is paired with protected time, visible leadership, accessible mentorship and research infrastructure.

Future studies should therefore extend follow‐up to approximately 12–24 months to determine whether changes in research literacy and research engagement are sustained and whether early project milestones subsequently lead to project completion, research funding, manuscript submission and publication.

### 4.5. Objective Progression and Real‐World Relevance

The project records add practical context to the questionnaire findings, but they require cautious interpretation. All intervention participants completed protocols and obtained ethics approval, almost all reached registration and data collection, and a substantial proportion progressed to writing and submission. Government‐funded projects and accepted or published manuscripts also occurred during the programme period. These milestones show what was achieved within the supported cohort, not what was caused by the programme.

Part of this progression may reflect the intervention, but part may also reflect baseline project maturity and the selection process itself. Projects were chosen partly on scientific merit, innovation and feasibility, characteristics that could independently increase the likelihood of reaching ethics approval, funding or publication. In addition, the comparison group did not have equivalent project‐progression data. The objective indicators are therefore descriptive and cannot provide a controlled estimate of programme effect. Longer follow‐up would also be needed to assess whether early milestones translate into sustained research activity and publication.

### 4.6. Strengths and Limitations

Strengths include implementation in routine clinical practice, a concurrent comparison group, baseline‐adjusted analysis, multiplicity control for the four core outcomes and objective project tracking. The intervention was also described with sufficient detail to show its intensity, delivery and tailoring.

Several limitations are important. First, allocation was based on formal multidisciplinary project review rather than randomisation. Because scientific merit, innovation and feasibility informed selection, the intervention group may have entered with stronger projects, greater research readiness or more favourable conditions for progression. These factors were not fully captured by the measured baseline covariates, and ANCOVA cannot remove residual or unmeasured confounding. Project‐review scores were not included in the analytic models. This concern is especially important for the descriptive project‐progression outcomes, for which baseline project maturity could itself influence ethics approval, funding, manuscript submission and publication. Second, the sample comprised the complete eligible annual cohort rather than a prospectively powered sample. With only 44 nurses, estimates are imprecise and sensitive to model specification. Third, the CRPL‐BSN had expert content review, pilot testing and generally acceptable internal consistency, but its factor structure and construct validity have not been established in an independent sample. Domain‐level changes should therefore be interpreted as preliminary and require replication using independently validated measures. Fourth, the study was conducted in one hospital. Organisational research culture, access to research administration, staffing and leadership support may differ elsewhere, limiting transferability to other settings. Fifth, both groups shared the same active research environment, which may have reduced between‐group contrast. Sixth, follow‐up ended after 7 months. This period may capture changes in research literacy and early project milestones but is too short to establish the durability of research behaviour or distal outcomes such as funding and publication. Seventh, the type, intensity and duration of participants’ previous research education and research experience were not standardised or quantitatively measured, which may have contributed to unmeasured heterogeneity in baseline research preparation. Finally, comparable objective progression data were unavailable for the comparison group, so these milestones cannot be attributed causally to the programme.

Future multicentre studies should undertake independent psychometric evaluation of the CRPL‐BSN in larger and more diverse samples, including examination of its dimensional structure and construct validity across settings, before it is considered for use as a standardised instrument.

## 5. Conclusion

In this exploratory nonrandomised evaluation, project‐embedded support was associated most consistently with interactive research literacy. Baseline‐adjusted differences in the other core domains were less robust after additional covariate adjustment. Objective project progression was observed within the intervention group but cannot be attributed to the programme without comparable control data. Larger, prospectively powered multicentre studies using independently validated measures, comparable objective outcomes and longer follow‐up are needed to test the sustainability and effectiveness of this approach.

## 6. Implications for Nursing Management

For nurse managers, the findings support considering research capacity as an organisational development issue rather than only an education issue. Repeated research dialogue, methodological consultation and longitudinal project tracking may help connect learning with real clinical problems, but these supports should be combined with protected time, leadership support and accessible research infrastructure. Future programmes should also build evaluation into implementation, including comparable objective milestones, longer follow‐up and designs that reduce selection bias before effectiveness is assumed.

Implementation should also be considered in relation to existing organisational resources. In this study, the PhD‐prepared nurse researcher was not an additional programme‐funded post but an existing Nursing Department nurse manager whose routine responsibilities included nursing research management, research training, clinical research project support and postgraduate nurse development. The research group, research consultations and longitudinal project tracking therefore represented a structured integration of existing research‐management functions. Over seven months, the programme involved six structured group activities, 47 project‐specific consultations and continuing project progress review. Although this suggests that the model can be embedded within an established nursing research‐management role, total staff time, participating nurses’ opportunity costs and other economic indicators were not systematically collected; cost‐effectiveness therefore cannot be inferred from this study.

Transferability may be more challenging in community or nonteaching hospitals with limited access to research mentors, statistical and methodological expertise, research administration or protected research time. In such settings, adaptations could include regional or cross‐institutional research mentors, academic–practice partnerships, group‐based research consultation or remote methodological support. These adaptations should be evaluated for feasibility, resource requirements and outcomes before broader implementation across different organisational contexts.

## Author Contributions

Qian Li: conceptualisation, methodology, intervention implementation, investigation, formal analysis, project administration, funding acquisition and writing–original draft. Ailing Ni: methodological input, interpretation of findings and writing–review and editing.

## Funding

Qian Li was supported by the Social Development Science and Technology Programme (Basic Research) of the Zhenjiang Science and Technology Bureau, China (Grant No. JC2025047).

## Disclosure

Both authors approved the final manuscript.

## Conflicts of Interest

The authors declare no conflicts of interest.

## Supporting Information

Additional supporting information can be found online in the Supporting Information section.

## Supporting information


**Supporting Information** Supporting Information includes five supporting tables: Table S1, theory‐to‐intervention mapping; Table S2, outcome domains and scoring structure of the CRPL‐BSN; Table S3, internal consistency and change‐score distribution checks; Table S4, adjusted intervention effects on theory‐informed secondary outcomes; and Table S5, objective research progression among intervention participants.

## Data Availability

Deidentified data that support the findings of this study are available from the corresponding author upon reasonable request, subject to institutional and ethical requirements.
