# Supplementary material for: Research Literacy and Research Engagement Following Project‐Embedded Support Among Clinical Nurses: An Exploratory Nonrandomised Controlled Study
Source: J Nurs Manag. 2026 Oct 21;2026:6444368. doi: 10.1155/jonm/6444368 (PMC13686408; doi:10.1155/jonm/6444368)
Supplement: Supplementary file 1 [file JONM-2026-6444368-s001.docx]

Supporting Information

Project-embedded support for clinical nurses’ research capacity: an exploratory non-randomised controlled study

**Table S1. Theory-to-intervention mapping**

| **Intervention target** | **Research-literacy target / COM-B enabling condition** | **Main barrier addressed** | **Intervention strategies** |
| --- | --- | --- | --- |
| Research understanding and question formation | Functional research literacy / Capability | Methods difficult to understand; clinical problems difficult to convert into researchable questions | Structured learning; question refinement; methodological guidance |
| Research dialogue and participation | Interactive research literacy / Capability + Opportunity | Limited opportunities to express, discuss and collaborate on research | Research salons; workshops; project discussion; peer and expert feedback |
| Critical judgement of value and feasibility | Critical research literacy / Capability + Motivation | Difficulty judging evidence quality, clinical meaning and feasibility | Evidence discussion; feasibility appraisal; protocol review |
| Translation from clinical problem to research action | Capability + Opportunity | Ideas or protocols fail to move into implementation | Study design; ethics and registration guidance; implementation support |
| Sustained project progression | Opportunity + Motivation | Methodological barriers, workload or discontinuity of support | Research clinic; stage tracking; individual consultation and feedback |

Note. Research-literacy targets and COM-B enabling conditions are complementary rather than one-to-one constructs.

**Table S2. Outcome domains and scoring structure of the CRPL-BSN**

| **Construct** | **Framework** | **Item set** | **Scoring** |
| --- | --- | --- | --- |
| Functional research literacy | Research literacy | F1–F10 | Mean; reverse F5, F8, F10 |
| Interactive research literacy | Research literacy | I1–I8 | Mean; reverse I5, I6, I7 |
| Critical research literacy | Research literacy | Cr1–Cr8 | Mean; reverse Cr6 |
| Research translation capability | COM-B: Capability | Ca1–Ca7 | Mean; reverse Ca3 |
| Research opportunity/support | COM-B: Opportunity | Op1–Op7 | Mean; reverse Op4, Op5, Op7 |
| Value-oriented research motivation | COM-B: Motivation | M1–M4, M8 | Mean |
| Research participation behaviour | Behaviour | BR1–BR6 | Mean |
| Evidence-use behaviour | Behaviour | BE1–BE5 | Mean |
| Lower formalistic research behaviour | Behaviour | BF1–BF5 | All items reverse-scored; higher = lower formalistic behaviour |
| Research persistence/development | Motivation/Behaviour | BP1–BP10 | Mean |

Note. All retained items used a 1–5 Likert response scale. Domain scores were the mean of the included items after reverse scoring where applicable. The revised manuscript reports the prespecified domain scores and does not use an overall composite score. CRPL-BSN = Clinical Research and Practice Literacy–Behavior Scale for Nurses.

**Table S3. Internal consistency and change-score distribution checks**

| **Outcome** | **Items** | **Cronbach’s α baseline** | **Cronbach’s α post** | **Shapiro–Wilk P: intervention change** | **Shapiro–Wilk P: comparison change** |
| --- | --- | --- | --- | --- | --- |
| Functional research literacy | 10 | 0.814 | 0.702 | 0.709 | 0.193 |
| Interactive research literacy | 8 | 0.771 | 0.710 | 0.586 | 0.762 |
| Critical research literacy | 8 | 0.736 | 0.691 | 0.658 | 0.184 |
| Research translation capability | 7 | 0.752 | 0.763 | 0.232 | 0.290 |
| Research opportunity/support | 7 | 0.741 | 0.719 | 0.742 | 0.303 |
| Value-oriented research motivation | 5 | 0.806 | 0.823 | 0.545 | 0.053 |
| Research participation behaviour | 6 | 0.901 | 0.899 | 0.149 | 0.123 |
| Evidence-use behaviour | 5 | 0.878 | 0.876 | 0.936 | 0.625 |
| Lower formalistic research behaviour | 5 | 0.806 | 0.882 | 0.804 | 0.070 |
| Research persistence/development | 10 | 0.924 | 0.938 | 0.112 | 0.634 |

Note. Cronbach’s alpha was calculated separately using all 44 participants at baseline and all 44 participants post-intervention. Shapiro–Wilk tests refer to within-group pre–post change scores.

**Table S4. Objective research progression among intervention participants**

| **Research milestone** | **Objective progression** |
| --- | --- |
| Research protocol completed | 19/19 (100.0%) |
| Ethics application submitted and approved | 19/19 (100.0%) |
| Study registration completed | 18/19 (94.7%) |
| Data collection commenced | 18/19 (94.7%) |
| Government-funded or grant-supported projects | 10 projects |
| Participants who commenced manuscript writing | 11/19 (57.9%) |
| Participants who submitted a manuscript | 9/19 (47.4%) |
| Science Citation Index (SCI)-indexed manuscripts published or accepted | 2 manuscripts |

*Note. Participant-level percentages use n=19 as the denominator. Funding is reported at the project level and publication status at the manuscript level. Milestones are not mutually exclusive and occurred within February–August 2026.*

**Table S5. Adjusted between-group differences on theory-informed secondary outcomes**

| Outcome | Main ANCOVA adjusted difference (95% CI) | P value | Partial η² | Sensitivity model adjusted difference (95% CI) | P value | Partial η² |
| --- | --- | --- | --- | --- | --- | --- |
| Research opportunity/support | 0.211 (−0.125, 0.547) | 0.211 | 0.038 | 0.015 (−0.304, 0.333) | 0.925 | <0.001 |
| Value-oriented research motivation | 0.310 (0.016, 0.604) | 0.040 | 0.099 | 0.156 (−0.137, 0.449) | 0.289 | 0.029 |
| Research participation behaviour | 0.451 (0.038, 0.864) | 0.033 | 0.106 | 0.220 (−0.180, 0.621) | 0.273 | 0.031 |
| Evidence-use behaviour | 0.336 (0.043, 0.630) | 0.026 | 0.115 | 0.216 (−0.087, 0.520) | 0.158 | 0.050 |
| Lower formalistic research behaviour | 0.512 (−0.066, 1.091) | 0.081 | 0.072 | 0.452 (−0.181, 1.086) | 0.157 | 0.051 |
| Research persistence/development | 0.415 (0.112, 0.718) | 0.009 | 0.157 | 0.292 (−0.028, 0.612) | 0.073 | 0.080 |

Note. The main ANCOVA adjusted for the corresponding baseline outcome score. The sensitivity model additionally adjusted for postgraduate education and senior professional title. Adjusted differences represent intervention minus concurrent comparison. These outcomes were treated as theory-informed secondary outcomes and were not included in the FDR correction applied to the four core outcomes.
